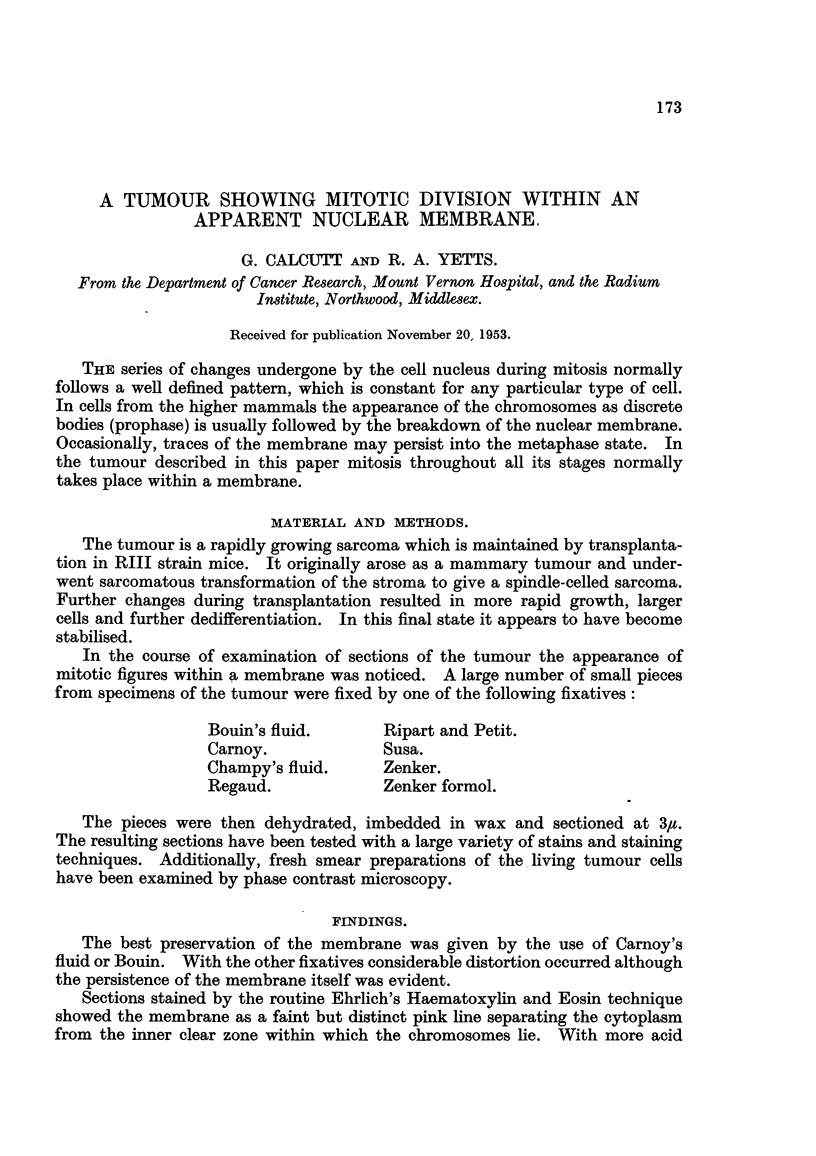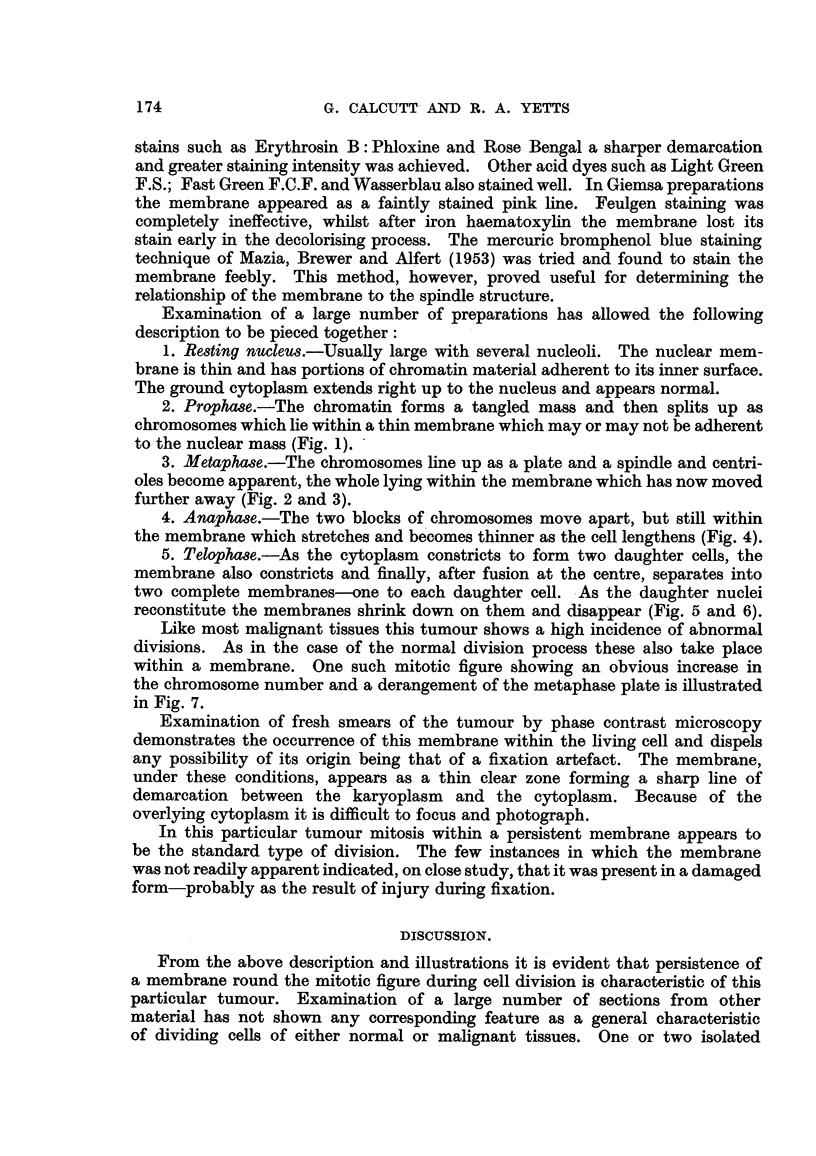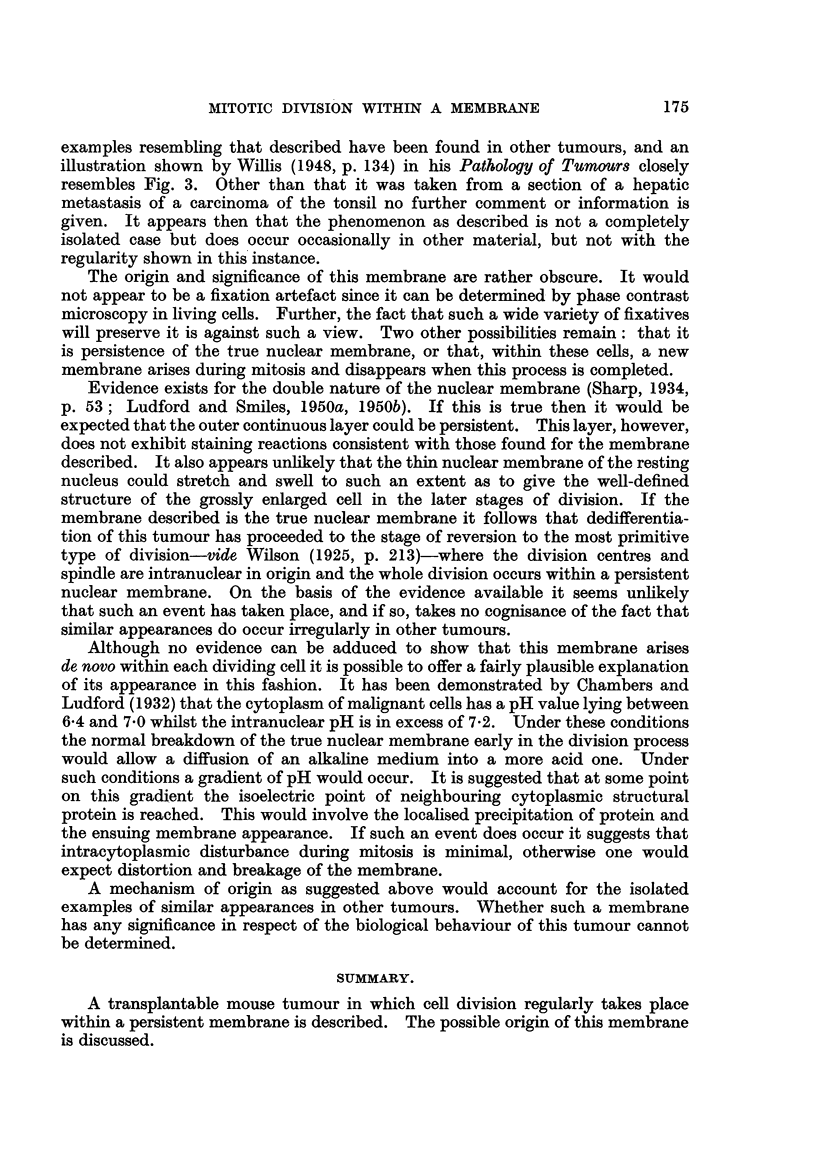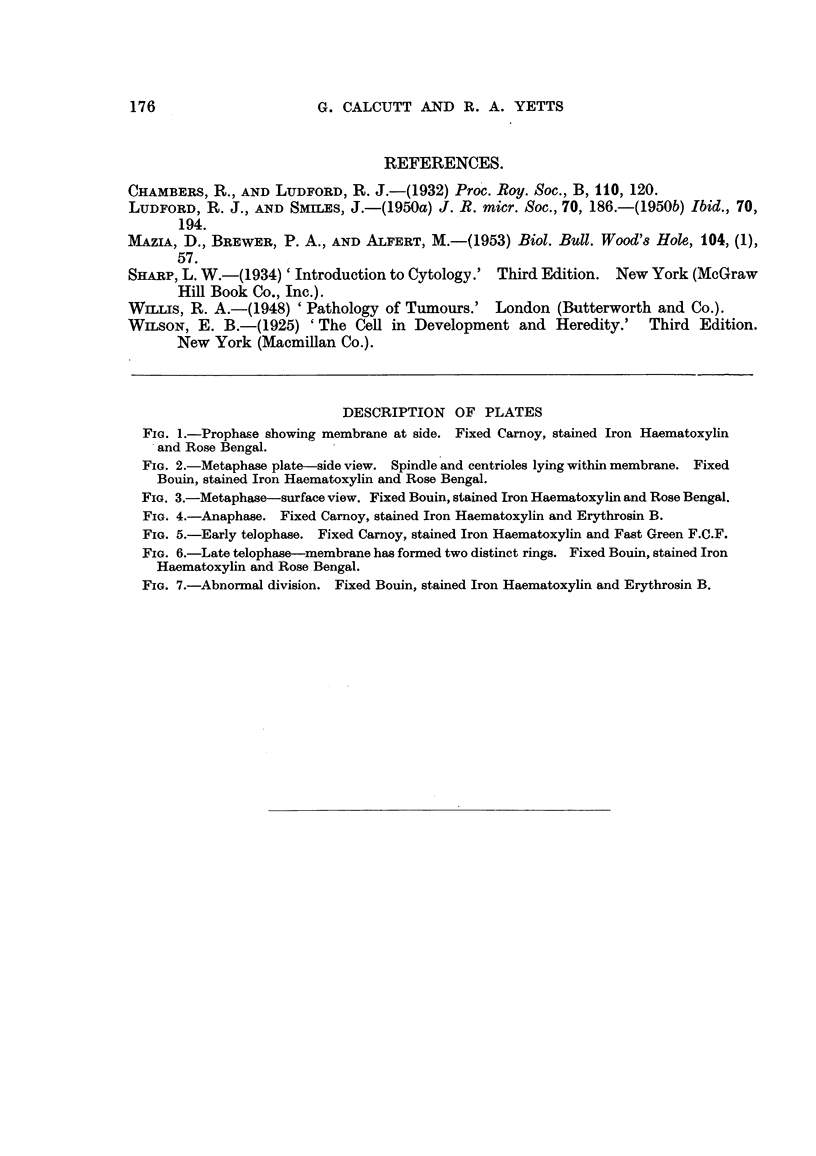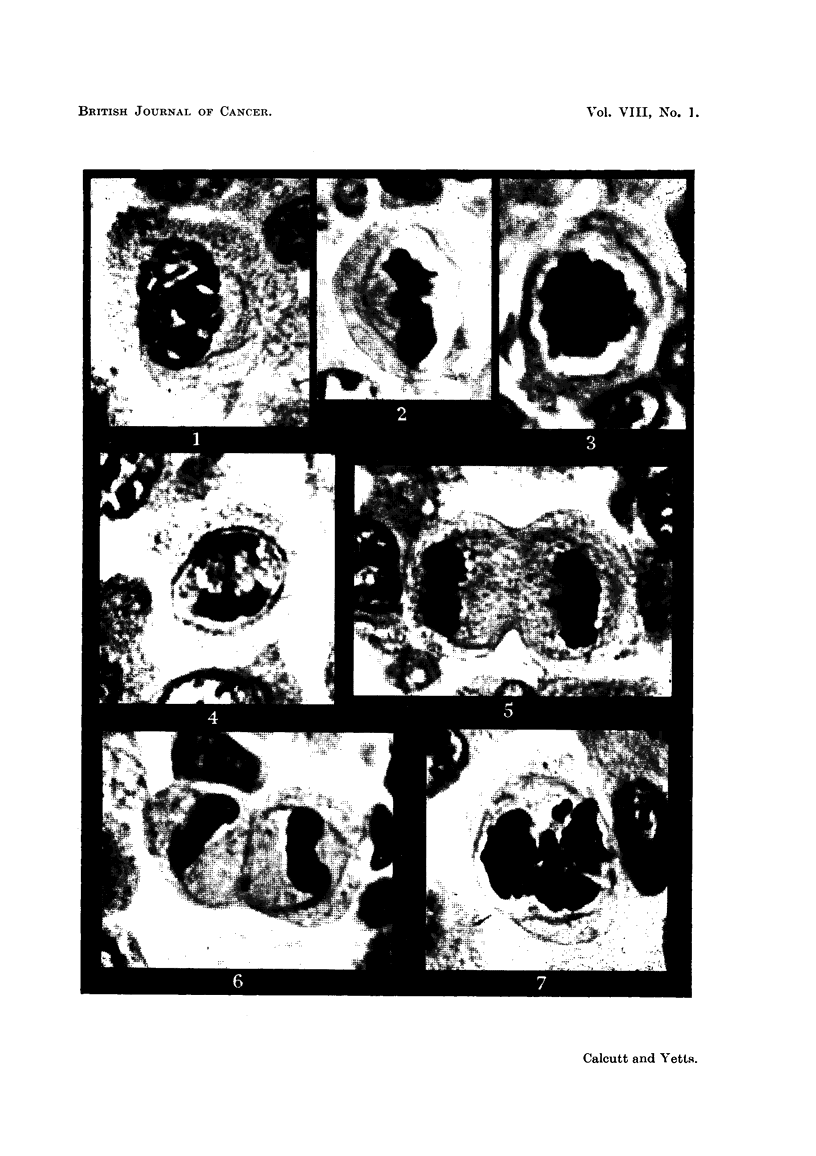# A Tumour Showing Mitotic Division within an Apparent Nuclear Membrane

**DOI:** 10.1038/bjc.1954.16

**Published:** 1954-03

**Authors:** G. Calcutt, R. A. Yetts

## Abstract

**Images:**


### A TUMOUR SHOWING MITOTIC DIVISION WITHIN AN APPARENT NUCLEAR MEMBRANE.

G. CALCUTT AND R. A. YETTS.

*From the Department of Cancer Research, Mount Vernon Hospital, and the Radium Institute, Northwood, Middlesex.*

Received for publication November 20, 1953.

THE series of changes undergone by the cell nucleus during mitosis normally follows a well defined pattern, which is constant for any particular type of cell. In cells from the higher mammals the appearance of the chromosomes as discrete bodies (prophase) is usually followed by the breakdown of the nuclear membrane. Occasionally, traces of the membrane may persist into the metaphase state. In the tumour described in this paper mitosis throughout all its stages normally takes place within a membrane.

### MATERIAL AND METHODS.

The tumour is a rapidly growing sarcoma which is maintained by transplantation in RIII strain mice. It originally arose as a mammary tumour and underwent sarcomatous transformation of the stroma to give a spindle-celled sarcoma. Further changes during transplantation resulted in more rapid growth, larger cells and further dedifferentiation. In this final state it appears to have become stabilised.

In the course of examination of sections of the tumour the appearance of mitotic figures within a membrane was noticed. A large number of small pieces from specimens of the tumour were fixed by one of the following fixatives :

| | |
|---|---|
| Bouin's fluid. | Ripart and Petit. |
| Carnoy. | Susa. |
| Champy's fluid. | Zenker. |
| Regaud. | Zenker formol. |

The pieces were then dehydrated, imbedded in wax and sectioned at $3\mu$. The resulting sections have been tested with a large variety of stains and staining techniques. Additionally, fresh smear preparations of the living tumour cells have been examined by phase contrast microscopy.

### FINDINGS.

The best preservation of the membrane was given by the use of Carnoy's fluid or Bouin. With the other fixatives considerable distortion occurred although the persistence of the membrane itself was evident.

Sections stained by the routine Ehrlich's Haematoxylin and Eosin technique showed the membrane as a faint but distinct pink line separating the cytoplasm from the inner clear zone within which the chromosomes lie. With more acid stains such as Erythrosin B : Phloxine and Rose Bengal a sharper demarcation and greater staining intensity was achieved. Other acid dyes such as Light Green F.S.; Fast Green F.C.F. and Wasserblau also stained well. In Giemsa preparations the membrane appeared as a faintly stained pink line. Feulgen staining was completely ineffective, whilst after iron haematoxylin the membrane lost its stain early in the decolorising process. The mercuric bromphenol blue staining technique of Mazia, Brewer and Alfert (1953) was tried and found to stain the membrane feebly. This method, however, proved useful for determining the relationship of the membrane to the spindle structure.

Examination of a large number of preparations has allowed the following description to be pieced together :

1. *Resting nucleus.*—Usually large with several nucleoli. The nuclear membrane is thin and has portions of chromatin material adherent to its inner surface. The ground cytoplasm extends right up to the nucleus and appears normal.

2. *Prophase.*—The chromatin forms a tangled mass and then splits up as chromosomes which lie within a thin membrane which may or may not be adherent to the nuclear mass (Fig. 1).

3. *Metaphase.*—The chromosomes line up as a plate and a spindle and centrioles become apparent, the whole lying within the membrane which has now moved further away (Fig. 2 and 3).

4. *Anaphase.*—The two blocks of chromosomes move apart, but still within the membrane which stretches and becomes thinner as the cell lengthens (Fig. 4).

5. *Telophase.*—As the cytoplasm constricts to form two daughter cells, the membrane also constricts and finally, after fusion at the centre, separates into two complete membranes—one to each daughter cell. As the daughter nuclei reconstitute the membranes shrink down on them and disappear (Fig. 5 and 6).

Like most malignant tissues this tumour shows a high incidence of abnormal divisions. As in the case of the normal division process these also take place within a membrane. One such mitotic figure showing an obvious increase in the chromosome number and a derangement of the metaphase plate is illustrated in Fig. 7.

Examination of fresh smears of the tumour by phase contrast microscopy demonstrates the occurrence of this membrane within the living cell and dispels any possibility of its origin being that of a fixation artefact. The membrane, under these conditions, appears as a thin clear zone forming a sharp line of demarcation between the karyoplasm and the cytoplasm. Because of the overlying cytoplasm it is difficult to focus and photograph.

In this particular tumour mitosis within a persistent membrane appears to be the standard type of division. The few instances in which the membrane was not readily apparent indicated, on close study, that it was present in a damaged form—probably as the result of injury during fixation.

### DISCUSSION.

From the above description and illustrations it is evident that persistence of a membrane round the mitotic figure during cell division is characteristic of this particular tumour. Examination of a large number of sections from other material has not shown any corresponding feature as a general characteristic of dividing cells of either normal or malignant tissues. One or two isolated examples resembling that described have been found in other tumours, and an illustration shown by Willis (1948, p. 134) in his *Pathology of Tumours* closely resembles Fig. 3. Other than that it was taken from a section of a hepatic metastasis of a carcinoma of the tonsil no further comment or information is given. It appears then that the phenomenon as described is not a completely isolated case but does occur occasionally in other material, but not with the regularity shown in this instance.

The origin and significance of this membrane are rather obscure. It would not appear to be a fixation artefact since it can be determined by phase contrast microscopy in living cells. Further, the fact that such a wide variety of fixatives will preserve it is against such a view. Two other possibilities remain: that it is persistence of the true nuclear membrane, or that, within these cells, a new membrane arises during mitosis and disappears when this process is completed.

Evidence exists for the double nature of the nuclear membrane (Sharp, 1934, p. 53; Ludford and Smiles, 1950*a*, 1950*b*). If this is true then it would be expected that the outer continuous layer could be persistent. This layer, however, does not exhibit staining reactions consistent with those found for the membrane described. It also appears unlikely that the thin nuclear membrane of the resting nucleus could stretch and swell to such an extent as to give the well-defined structure of the grossly enlarged cell in the later stages of division. If the membrane described is the true nuclear membrane it follows that dedifferentiation of this tumour has proceeded to the stage of reversion to the most primitive type of division—*vide* Wilson (1925, p. 213)—where the division centres and spindle are intranuclear in origin and the whole division occurs within a persistent nuclear membrane. On the basis of the evidence available it seems unlikely that such an event has taken place, and if so, takes no cognisance of the fact that similar appearances do occur irregularly in other tumours.

Although no evidence can be adduced to show that this membrane arises *de novo* within each dividing cell it is possible to offer a fairly plausible explanation of its appearance in this fashion. It has been demonstrated by Chambers and Ludford (1932) that the cytoplasm of malignant cells has a pH value lying between 6·4 and 7·0 whilst the intranuclear pH is in excess of 7·2. Under these conditions the normal breakdown of the true nuclear membrane early in the division process would allow a diffusion of an alkaline medium into a more acid one. Under such conditions a gradient of pH would occur. It is suggested that at some point on this gradient the isoelectric point of neighbouring cytoplasmic structural protein is reached. This would involve the localised precipitation of protein and the ensuing membrane appearance. If such an event does occur it suggests that intracytoplasmic disturbance during mitosis is minimal, otherwise one would expect distortion and breakage of the membrane.

A mechanism of origin as suggested above would account for the isolated examples of similar appearances in other tumours. Whether such a membrane has any significance in respect of the biological behaviour of this tumour cannot be determined.

### SUMMARY.

A transplantable mouse tumour in which cell division regularly takes place within a persistent membrane is described. The possible origin of this membrane is discussed.

### REFERENCES.

CHAMBERS, R., AND LUDFORD, R. J.—(1932) *Proc. Roy. Soc.*, B, **110**, 120.

LUDFORD, R. J., AND SMILES, J.—(1950*a*) *J. R. micr. Soc.*, **70**, 186.—(1950*b*) *Ibid.*, **70**, 194.

MAZIA, D., BREWER, P. A., AND ALFERT, M.—(1953) *Biol. Bull. Wood's Hole*, **104**, (1), 57.

SHARP, L. W.—(1934) ' Introduction to Cytology.' Third Edition. New York (McGraw Hill Book Co., Inc.).

WILLIS, R. A.—(1948) ' Pathology of Tumours.' London (Butterworth and Co.).

WILSON, E. B.—(1925) ' The Cell in Development and Heredity.' Third Edition. New York (Macmillan Co.).

---

### DESCRIPTION OF PLATES

FIG. 1.—Prophase showing membrane at side. Fixed Carnoy, stained Iron Haematoxylin and Rose Bengal.

FIG. 2.—Metaphase plate—side view. Spindle and centrioles lying within membrane. Fixed Bouin, stained Iron Haematoxylin and Rose Bengal.

FIG. 3.—Metaphase—surface view. Fixed Bouin, stained Iron Haematoxylin and Rose Bengal.

FIG. 4.—Anaphase. Fixed Carnoy, stained Iron Haematoxylin and Erythrosin B.

FIG. 5.—Early telophase. Fixed Carnoy, stained Iron Haematoxylin and Fast Green F.C.F.

FIG. 6.—Late telophase—membrane has formed two distinct rings. Fixed Bouin, stained Iron Haematoxylin and Rose Bengal.

FIG. 7.—Abnormal division. Fixed Bouin, stained Iron Haematoxylin and Erythrosin B.